# Stable Expression of a Hepatitis E Virus (HEV) RNA Replicon in Two Mammalian Cell Lines to Assess Mechanism of Innate Immunity and Antiviral Response

**DOI:** 10.3389/fmicb.2020.603699

**Published:** 2020-12-03

**Authors:** Ling-Dong Xu, Fei Zhang, Lei Peng, Wen-Ting Luo, Chu Chen, Pinglong Xu, Yao-Wei Huang

**Affiliations:** ^1^Institute of Preventive Veterinary Science and Key Laboratory of Animal Virology of Ministry of Agriculture, Department of Veterinary Medicine, Zhejiang University, Hangzhou, China; ^2^MOE Laboratory of Biosystems Homeostasis & Protection and Innovation Center for Cell Signaling Network, Life Sciences Institute, Zhejiang University, Hangzhou, China; ^3^Zhaoqing Branch Center of Guangdong Laboratory for Lingnan Modern Agricultural Science and Technology, Zhaoqing, China

**Keywords:** hepatitis E virus (HEV), replicon, antiviral drugs, interferon, innate immune sensing

## Abstract

Hepatitis E virus (HEV) is one of the major etiological agents responsible for acute hepatitis. Hepatitis E virus does not replicate efficiently in mammalian cell cultures, thus a useful model that mimics persistent HEV replication is needed to dissect the molecular mechanism of pathogenesis. Here we report a genotype-3 HEV RNA replicon expressing an EGFP-Zeocin (EZ) resistant gene (p6-EZ) that persistently self-replicated in cell lines of human (Huh-7-S10-3) or hamster (BHK-21) origin after transfection with *in vitro* RNA transcripts and subsequent drug screening. Two cell lines, S10-3-EZ and BHK-21-EZ, stably expressed EGFP in the presence of Zeocin during continuous passages. Both genomic and subgenomic HEV RNAs and viral replicase proteins were stably expressed in persistent HEV replicon cells. The values of the cell models in antiviral testing, innate immune RNA sensing and type I IFN in host defense were further demonstrated. We revealed a role of RIG-I like receptor-interferon regulatory factor 3 in host antiviral innate immune sensing during HEV replication. We further demonstrated that treatment with interferon (IFN-α) or ribavirin significantly reduced expression of replicon RNA in a dose-dependent manner. The availability of the models will greatly facilitate HEV-specific antiviral development, and delineate mechanisms of HEV replication.

## Introduction

Hepatitis E virus (HEV), a major cause of acute viral hepatitis in humans, is an important public health concern, causing approximately 57,000 hepatitis E-related deaths each year (Meng, 2016). Although the overall mortality associated with HEV infection is low with less than 1% in the general population, it can reach up to 25% in infected pregnant women (Meng, 2016). In developing countries, genotypes 1 and 2 HEVs in the species *Orthohepevirus A* of the family Hepeviridae, are commonly transmitted by the fecal-oral route via virus-contaminated water often causing large outbreaks, while in industrialized countries, sporadic and cluster cases of hepatitis E are mostly caused by the zoonotic genotypes 3 and 4 HEVs (Meng, 2003; Johne et al., 2014; Meng, 2016). More recently, chronic hepatitis E with persistent genotype 3 HEV infection in immunosuppressed individuals such as organ transplant recipients (Péron et al., 2006; Legrand-Abravanel et al., 2010) and HIV-infected patients (Dalton et al., 2009; Fujiwara et al., 2014) has become a significant clinical problem, and required antiviral treatment.

Hepatitis E virus is a small, non-enveloped virus with a single-stranded, positive-sense RNA genome, though virions in the bloodstream may cloak themselves within a host cell membrane to produce quasi-enveloped virions (Takahashi et al., 2010; Yin et al., 2016; Nagashima et al., 2017). The HEV genome is approximately 7.2-kb in size, including short 5′ and 3′ non-coding regions and three open reading frames (ORFs; Emerson and Purcell, 2003). ORF1, located at the 5′ end of the viral genome, encodes the non-structural proteins that are involved in viral replication. At the 3′ end, ORF2 encodes a 660-amino acid (aa) capsid protein. ORF3, which almost completely overlaps ORF2, encodes a small 113-aa ion channel protein that is required for release of infectious particles (Huang et al., 2007; Ding et al., 2017). An intragenomic promoter has also been recently revealed that regulates subgenomic RNA synthesis (Ding et al., 2018). However, due to the lack of an efficient cell culture system mimicking persistent HEV infection and a small conventional animal model for HEV infection, our knowledges of HEV–host interaction and mechanism of HEV pathogenesis are still very limited.

Recently, with the isolation of strains of genotypes 3 and 4 HEVs from infected patients that can be propagated more efficiently *in vitro* (Tanaka et al., 2007, 2009), and with the discovery of a genotype 3 HEV strain with insertion of a 58-aa sequence from human ribosomal protein S17 that improved viral replication *in vitro* (Shukla et al., 2011), we now have limited but useful tools to study the HEV life cycle. However, major obstacles remain for HEV research. The existing HEV replicon systems such as HEV-GFP (green fluorescent protein; Emerson et al., 2004) and the genotype 3 HEV replicon (Kernow-C1 p6/gluc; Shukla et al., 2012) are unsuitable for antiviral screening since they cannot replicate continuously and stably in cells, and must be transcribed from infectious clones in every cycle. Therefore, an *in vitro* cell culture model mimicking persistent HEV infection is critically needed to screen HEV-specific antivirals and delineate the mechanism of HEV pathogenesis.

Here, we report the generation of a stable HEV RNA replicon system in both BHK-21 and S10-3 cells. Our replicon-bearing cells could stably expressed EGFP in the presence of Zeocin after multiple passages, with full-length replicon and a single subgenomic RNA detected by Northern blot. We further illustrated the unique value of the practical models, by demonstrating the importance of RNA innate immune sensing, as well as the effectiveness of antivirals including ribavirin, IFNα-2a, and siRNA, in limiting HEV infection. Therefore, the HEV replicon cell lines will greatly facilitate our understanding of mechanisms of HEV replication and aid in identifying an effective HEV-specific antiviral in the future.

## Materials and Methods

### Cells, Antibodies and Compounds

The Huh7-S10-3 cell line (a subclone of the human hepatoma cell line Huh-7) was a gift from Dr. Suzanne U. Emerson (NIH, Bethesda, MD, United States). The control S10-3-GFP and BHK-GFP stable cell lines with GFP overexpression were generated by the lentivirus expression system. BHK-21 and Huh7-S10-3 cells were maintained in Dulbecco’s minimal essential medium (DMEM) containing 10% fetal bovine serum (Gibco), penicillin (250 IU/ml) and streptomycin (250 μg/ml) at 37°C in the presence of 5% CO_2_. Ribavirin, BX795, and MRT67307 were purchased from Selleck Chemicals, poly (I:C) from Invivogen, zeocin from Thermo Fisher and peg-IFNα-2a from Shanghai Roche Pharmaceuticals Co., Ltd. The anti-IRF3 (#4302S), anti-pIRF3 (#4947S), anti-TBK1 (#3504S), anti-pTBK1 (#5483S), and anti-HA (#3724S) antibodies were purchased from Cell Signaling Technology. The anti-α-tubulin (#T6199) antibodies were purchased from Sigma and anti-GFP (#sc-8334) from Santa Cruz Biotechnology. The anti-dsRNA antibody (#J2-1702) was purchased from SCICONS (Hungry). The source of the rabbit anti-HEV-ORF2 antibody was previously described (Kenney and Meng, 2015), and the rabbit polyclonal antibody against HEV helicase domain (aa 856–1014) of ORF1 (GenBank protein ID: AFD33683) was generated in-house. The anti-X domain monoclonal antibody was a gift from Dr. Yonglin Yang (Nanjing Red Cross Blood Center, Nanjing, China), which has been described most recently (Ju et al., 2020).

### Construction of an HEV Replicon Plasmid

**FIGURE 1:**
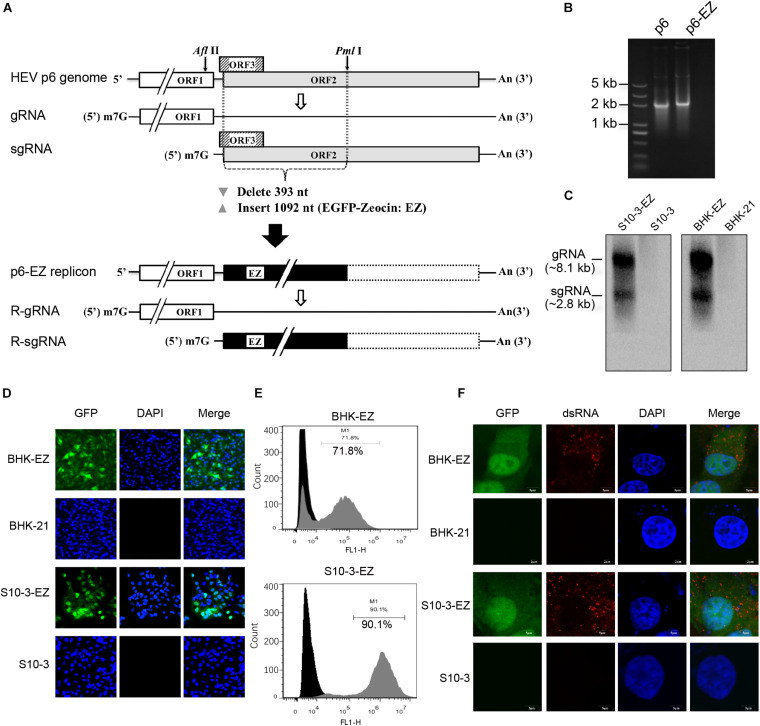
Establishment of two stable persistent HEV RNA replicon cell lines BHK-EZ and S10-3-EZ. **(A)** A schematic diagram of HEV replicon p6-EZ. The HEV ORF1 is intact, whereas only the first 11 nt of the ORF3 remains; the first 393 nt of the ORF2 are replaced by a gene (1,092 nt) encoding EGFP-zeocin (EZ) followed by a termination codon. Open rectangles: HEV coding regions; black rectangle: EGFP/zeocin (EZ) coding region; dashed-line rectangle: residual ORF2 gene (not translated). **(B)** Agarose gel electrophoresis showing the transcribed RNAs of p6 and p6-EZ clones. **(C)** Northern blot analyses of positive-strand viral RNA detected in HEV persistent replicon cell lines S10-3-EZ and BHK-EZ. Total RNAs extracted from BHK-EZ and S10-3-EZ and their respective parental cell lines were probed with RNA specific to the 7182–7240 nt fragment of p6-EZ. The upper bands are full-length p6-EZ RNAs, and the lower bands are subgenomic RNAs. **(D)** The expression of EGFP was observed by fluorescence microscopy in S10-3-EZ and BHK-EZ cells. **(E)** Detection of GFP-positive cell rate of HEV persistent replicon cell lines S10-3-EZ and BHK-EZ by FACS. **(F)** Immunofluorescence (IFA) of S10-3-EZ and BHK-EZ or respective parental cell lines, stained with anti-dsRNA monoclonal antibody, to confirm viral replication.

By utilizing the backbone of the genotype 3 human HEV Kernow-C1/p6 infectious cDNA clone which contains the full-length HEV genome (GenBank accession number JQ679013; Shukla et al., 2011), we constructed the HEV p6-EZ replicon in this study by fusion PCR containing the 3′ region of ORF1 fused to the EGFP/zeocin gene, and subcloned between *Afl*II and *Pml*I restriction sites in the infectious cDNA clone backbone (Figure 1A). The ORF1 region from nt 4765–5358 was amplified by PCR from the infectious cDNA clone using the forward primer AflII594-F (5′-GATGTCTCTTAAGGGTTTCTGGAAGAAGCATTC-3′) and the reverse primer AflII594-R/EGFP/zeocin (5′-CCC TTGCTCACCATGGTGATCCCATGGGCGATGC-3′). The EZ gene was amplified from pTracer-CMV (Invitrogen) using the forward primer ORF2-EZ-F (5′-GCCA TGGGATCACCATGGTGAGCAAGGGCGAGGA-3′) and the reverse primer *Pml*I-EZ-R (5′-CACCACGTGAATCA GTCCTGCTCCTCGGCCACGAA-3′; the introduced stop codons are underlined). All PCR products were purified with a GeneJET Gel Extraction Kit (Thermo Fisher Scientific); fusion PCR was performed by using the forward primer *Afl*II-594-F and the reverse primer *Pml*I-EZ-R, digested with *Afl*II and *Pml*I, and ligated into the large fragment of the infectious HEV cDNA clone from which the *Afl*II-*Pml*I region had been deleted. The sequence of the entire HEV replicon (p6-EZ) was verified by Sanger sequencing. The expression plasmids encoding HA-tagged human TBK1 have been described previously (Wu et al., 2019).

### Transcription, Transfection, and Selection of Zeocin-Resistant Cells

The plasmid p6-EZ was linearized with *Mlu*I (NEB), and 1 μg of linearized DNA was used as the template for *in vitro* transcription with the mMESSAGE mMACHINE T7 ULTRA Transcription Kit (Ambion) at 37°C for 90 min. Prior to transfection, cells in six-well plates were washed with Opti-MEM (Gibco), and 5 μl of the transcription mixture was added to 700 μl Opti-MEM containing 5 μl DMRIE-C per well. The transfected cells were incubated at 34.5°C. After 5 h incubation, the transfection mixture was aspirated, fresh medium added, and the cultures were incubated at 37°C. Zeocin was added to culture medium at a concentration of 250 μg/ml, and refreshed every two days. The selected cell lines were further sorted according to the GFP expression by fluorescence-activated cell sorting (FACS). The generated stable cell lines were maintained with culture medium containing zeocin (250 μg/ml).

### Immunofluorescence Assay (IFA) and Western Blot Analysis

Cells grown on slides were rinsed in PBS 3 times, and fixed with 80% acetone at **−**20°C for 20 min. Cells were then overlaid with a 1:1 mixture of 10% bovine serum albumin and PBS containing a mixture of anti-X domain HEV antibody for 1 h and treated with goat anti-mouse IgG conjugated with Alexa Fluor 594 (Invitrogen) for 1 h. After adding 4,6-diamidino-2-phenylindole (DAPI) mounting solution (Sigma), slides were viewed with a fluorescence microscope (Leica). Between every two steps, the cells were rinsed in PBS three times. Confocal fluorescent images were obtained with a confocal laser scanning microscope (Fluoviewver FV1000-IX81; Olympus, Japan). Western blot analysis was conducted as described previously (Zhang et al., 2017; Wu et al., 2019).

### Treatment of Persistent HEV Replicon Cells With siRNA

Four different siRNAs targeting the p6-EZ replicon were designed in this study: three of them were specific for RNA-dependent RNA polymerase, RdRp [siRdRp1 (5′-CCACAAGAGCUUACCGUGU-3′); siRdRp2 (5′-CCACUUUACAGAGACUAUU-3′); siRdRp3 (5′-GCUACGUCUUGCUGUUUGU-3′)], and the target of siGFP was the EGFP/zeocin gene. All siRNAs were purchased from RiboBio Co., Ltd. (Guangzhou, China), and were transfected using Lipofectamine RNAiMAX transfection reagent (Thermo Fisher Scientific) according to manufacturer’s instructions. The BHK-EZ HEV replicon cell line was analyzed for EGFP expression at 48 and 96 h post-treatment, and the level of EGFP expression was determined by fluorescence microscopy and FACS.

### Treatment of Persistent HEV Replicon Cells With Known HEV Antivirals and Inhibitors of Antiviral Immunity

The effect of peg-IFNα-2a, ribavirin, BX795, or MRT67307 on HEV replication in persistent HEV replicon-bearing cells was examined at concentrations ranging from 125 to 2,000 IU/ml, 0 to 45 μM, 0.1 to 10 μM, or 0.1 to 10 μM, respectively. Various concentrations of drugs were added to one day-old, 60–70% confluent BHK-EZ and S10-3-EZ HEV replicon cell cultures, respectively. EGFP expression of the persistent HEV replicon p6-EZ was examined by fluorescence microscopy and FACS. Fluorescence-activated cell sorting was used to assess the mean percentage of GFP-positive cells and the relative fluorescence intensity (RFI).

## Results

### Establishment of Two Practical Stable and Persistently Replicating HEV Replicon Cell Lines

To generate a stable HEV replicon, a genotype 3 human HEV infectious clone (Kernow-C1 p6; Shukla et al., 2012) was engineered by replacing the N-terminal part of ORF2 (including the C-terminus of ORF3) with an enhanced GFP (EGFP)-zeocin resistance hybrid gene (p6-EZ; Figure 1A; Bennett et al., 1998). Upon transfection of BHK-21 or Huh7-S10-3 cells with capped RNA transcripts derived from p6 or p6-EZ (Figure 1B), two stable HEV replicon cell lines, BHK-EZ and S10-3-EZ, were successfully generated via zeocin selection. Importantly, HEV RNA species in BHK-EZ and S10-3-EZ cells were reproducibly detected by Northern blot analysis with a digoxigenin-labeled probe, showing the presence of both genomic and subgenomic HEV RNAs (Figure 1C). By using fluorescence microscopy, GFP expression was observed in BHK-EZ or S10-3-EZ cells (Figure 1D). Furthermore, by using FACS analysis, we showed that the EGFP-positive HEV replicon cells reached a stable proportion of 72% in BHK-EZ cells and 90% in S10-3-EZ cells under zeocin selection (Figure 1E). We employed an IFA assay that detected dsRNA (Figure 1F), demonstrating the active HEV replicative state in persistent HEV replicon cell lines simultaneously displaying nuclear translocation of the EGFP fusion protein with the zeocin resistance marker as described previously (Bennett et al., 1998).

**FIGURE 2:**
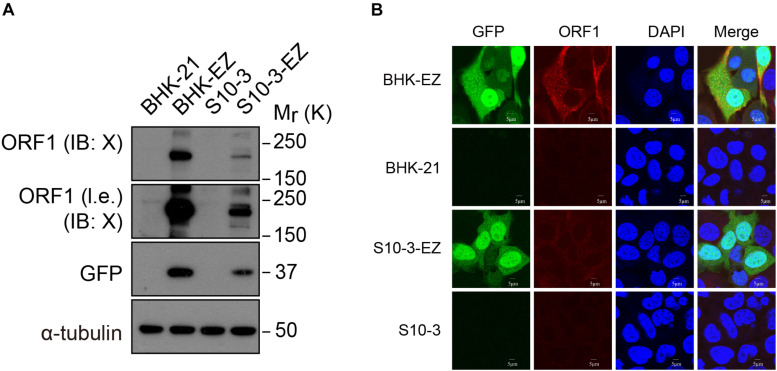
Detection of ORF1 expression in two HEV replicon cell lines. **(A)** Western blot analysis confirming EGFP and ORF1 protein expression in S10-3-EZ and BHK-EZ cells. **(B)** Cell lines were stained with anti-HEV ORF1 X domain monoclonal antibody to confirm ORF1 expression by IFA.

In the immunoblot analyses using a specific mouse monoclonal antibody against the X domain of ORF1, we also detected the expression of HEV ORF1 protein both in S10-3-EZ and in BHK-EZ HEV replicon cells (Figure 2A). Hepatitis E virus ORF1 co-localized with GFP fusion protein in the cytoplasm of BHK-EZ cells (Figure 2B). These results collectively illustrate the successful establishment of stable and replicating HEV replicon cell lines.

### Infectivity of RNA Isolated From Replicon-Bearing Cells and Identification the Stability of the Replicon Cell Lines

**FIGURE 3:**
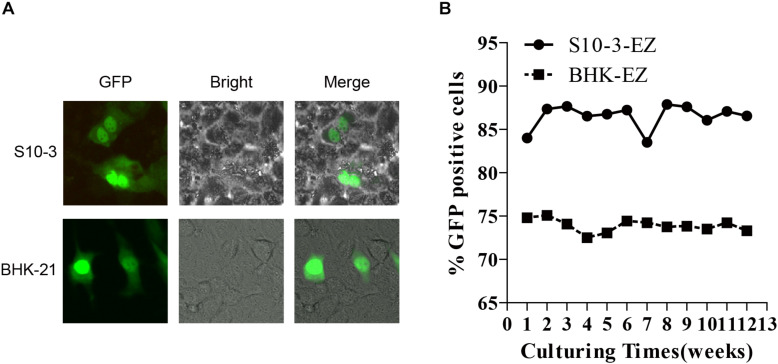
Stable and continuous expression of HEV RNA in persistent HEV replicon cell lines BHK-EZ and S10-3-EZ. **(A)** Total RNAs extracted from BHK-EZ and S10-3-EZ cells were used to transfect naïve BHK-21 and S10-3, resulting in green fluorescence signals that indicate the existence of replicating HEV genomes in cells in the form of RNA, and does not integrate into the host cell genome. **(B)** Detection of HEV replicon stability in the two cell lines by FACS.

Furthermore, we transfected the RNAs extracted from BHK-EZ and S10-3-EZ cells into naïve BHK-21 and S10-3 cells to assess their replication competency. As expected, the EGFP-positive cells were successfully detected at 72 h post-transfection (Figure 3A). The HEV genome in both the BHK-EZ and S10-3-EZ replicon cell lines can replicate stably with antibiotic selection for more than 12 weeks, maintaining p6-EZ replicon-positive without significant changes in EGFP expression (Figure 3B). Furthermore, a time-course qRT-PCR analysis targeting the ORF1 of HEV RNA in two stable cell lines demonstrated that the replication of HEV replicons were efficient and stable overtime during a 15-day culturing period (Supplementary Figure 1).

### The Persistent HEV Replicon Cell Lines Offer Unique Tools for Testing HEV Antivirals (IFNα, Ribavirin)

**FIGURE 4:**
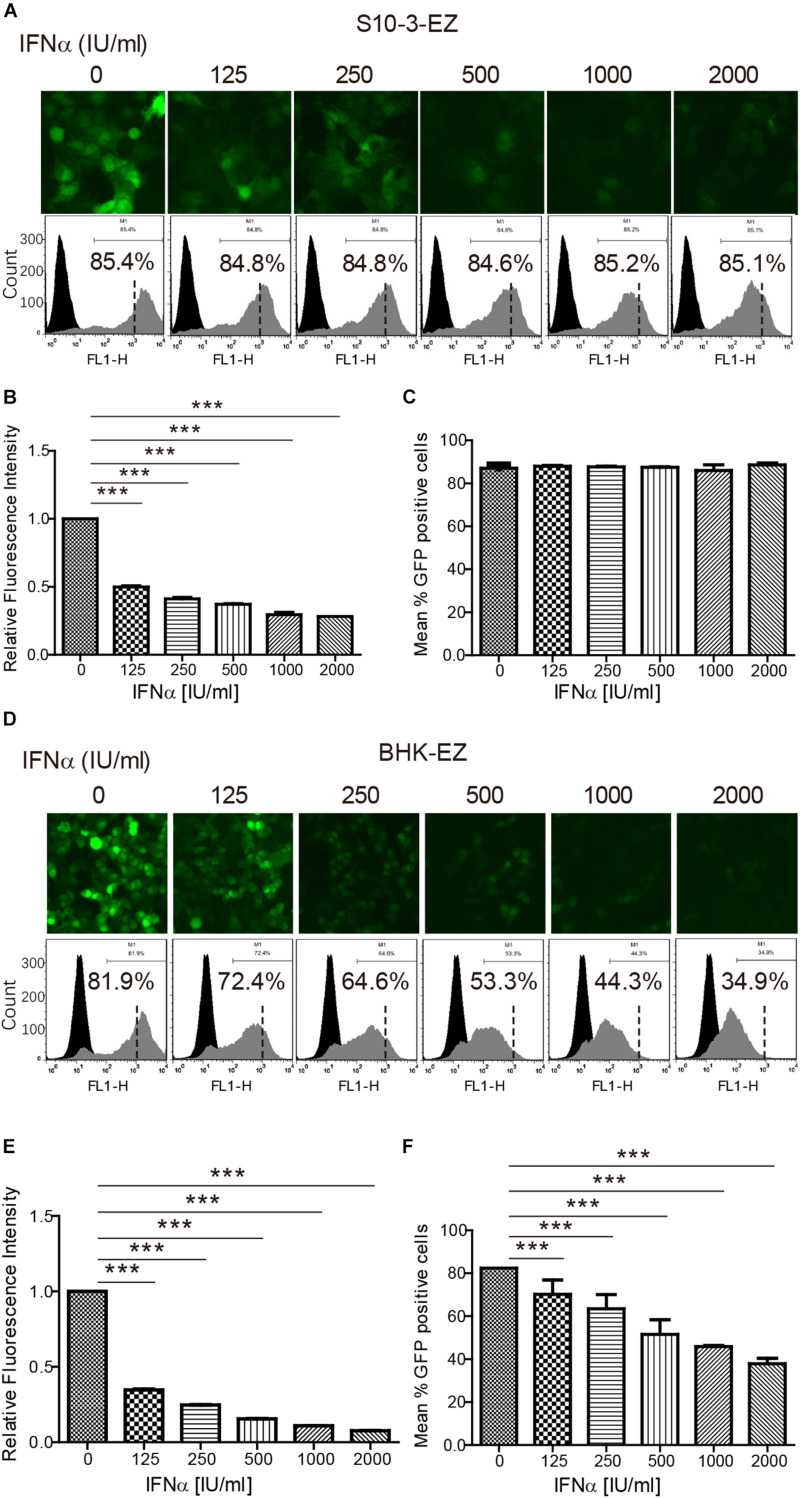
The persistent HEV replicon cell lines are invaluable in assessing HEV antiviral response. The persistent HEV replicon cell lines S10-3-EZ and BHK-EZ were treated with various concentrations (0–2,000 IU/ml) of peg-IFNα-2a for 48 h. **(A,D)** Observations with fluorescent microscope indicate a dose-dependent inhibition of GFP expression. **(B,E)** Quantitative FACS analysis confirming peg-IFNα-2a inhibition of GFP expression based on relative fluorescence intensity (RFI). **(C,F)** The number of GFP-positive cells was determined by FACS after peg-IFNα-2a treatment, shown as a percentage of total counted cells. Error bars indicate standard deviation; *P < 0.05; **P < 0.01; and ***P < 0.001.

To evaluate the usefulness of the two persistent HEV replicon cell lines for potential antiviral screening in the future, we determined the antiviral effects of two known anti-HEV drugs, pegylated IFNα (peg-IFNα-2a) and ribavirin, in BHK-EZ and S10-3-EZ HEV replicon cells. As controls, treatment of interferon or ribavirin had no effects on the GFP expression in the S10-3-GFP or BHK-GFP cell lines (Supplementary Figure 2). Concentrations of peg-IFNα-2a up to 2,000 IU/ml and ribavirin up to 45 μM showed no noticeable cytotoxic effects to the cells. EGFP expression in S10-3-EZ and BHK-EZ HEV replicon cells, indicative of transcription and expression of the viral genome, was monitored by fluorescence microscopy and FACS in both cell lines (Figures 4A,D). By calculating the percentage of GFP-positive cells and RFI, we showed that administration of either peg-IFNα-2a (Figures 4B,E) or ribavirin (Supplementary Figures 3A,C) effectively inhibited HEV replication in a dose-dependent manner. Relative fluorescence intensity was reduced by 48 h after treatments with both antivirals, with an ED_50_ (50% effective dose) of 125 IU/ml of peg-IFNα-2a and 15–25 μM of ribavirin. Treatment of BHK-EZ cells (but not S10-3-EZ cells) with peg-IFNα-2a for 48 h resulted in partial clearance of the replicating HEV genome, as evidenced by a decrease in GFP-positive cells (Figures 4C,F). Compared to the peg-IFNα-2a, effect to viral clearance is minimal upon ribavirin treatment in BHK-EZ cells, since the percentage decrease of GFP-positive cells is not significant (Figure 4F and Supplementary Figures 3B,D).

These observations confirm the known clinical observations that type I IFN and the broad-spectrum antiviral ribavirin are effective in suppression of HEV replication, thus proving that the persistent HEV replicon cell lines established in this study are valuable tool for future screening and discovery of HEV-specific antivirals.

### Effects of siRNA on the HEV Replicon Cell Lines

**FIGURE 5:**
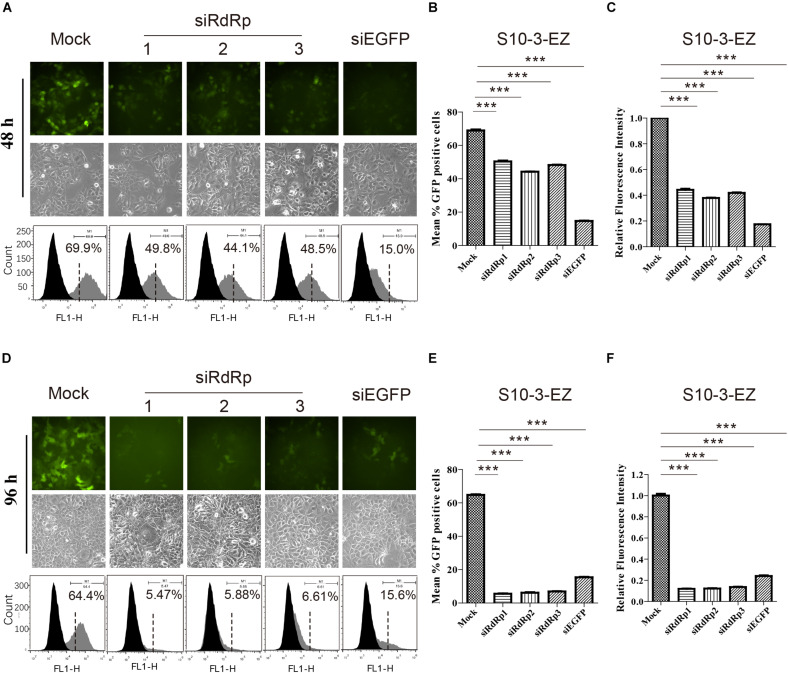
The effects of siRNA interference on persistent HEV replicon cell line S10-3-EZ. **(A,D)** S10-3-EZ cells were observed by fluorescence microscopy, and GFP-positive cells were counted by FACS 48 h or 96 h after siRNA treatment. **(B,E)** The number of GFP-positive cells as determined by FACS is shown as a percentage of total counted cells (hpt: hours post-transfection). **(C,F)** Quantitative FACS analysis confirming siRNA inhibition of GFP expression by RFI. GFP expression in cells treated with siRNA is shown as a relative percentage of the mock-treated control. Error bars indicate standard deviation; *P < 0.05; **P < 0.01; and ***P < 0.001.

We also examined the effects of siRNA interference on HEV replication in both S10-3-EZ and BHK-EZ cell lines. Three distinct siRNAs targeting the RdRp of HEV genome (siRdRp) were tested, and their effects in inhibiting HEV replication were monitored by fluorescence microscopy and FACS at 48 and 96 h post-transfection (Figures 5A,D and Supplementary Figures 4A,D). siRNA administration diminished the GFP-positive S10-3-EZ and BHK-EZ cells, with optimal effect reached after 96 h of treatment, indicating that the persistent HEV replicon is sensitive to siRNA interference (Figures 5A–F and Supplementary Figures 4A–F), and thus siRNA interference targeting the RdRp region of HEV genome may be an effective treatment for HEV infection.

Interestingly, siRNA targeting EGFP (siEGFP) showed strong effect against the EGFP expression at 48 h but not maintained until 96 h (Supplementary Figures 4A,D). We speculate that the knockdown effect of the three siRdRps were likely higher than that of siEGFP, since the EGFP expression was driven by the HEV subgenomic RNA instead of HEV genome replication. At 48 h, the EGFP expression was inhibited by siEGFP. The suppression was likely recovered at 96 h due to persistent production of subgenomic RNA by HEV replication, which probably overwhelmed siEGFP. In contrast, replication of HEV replicon was continuously suppressed by the siRdRp targeting the replicase gene ORF1.

### The Persistent HEV Replicon Cell Lines Afford an Opportunity to Study Host Antiviral Sensing and Response During HEV Replication

**FIGURE 6:**
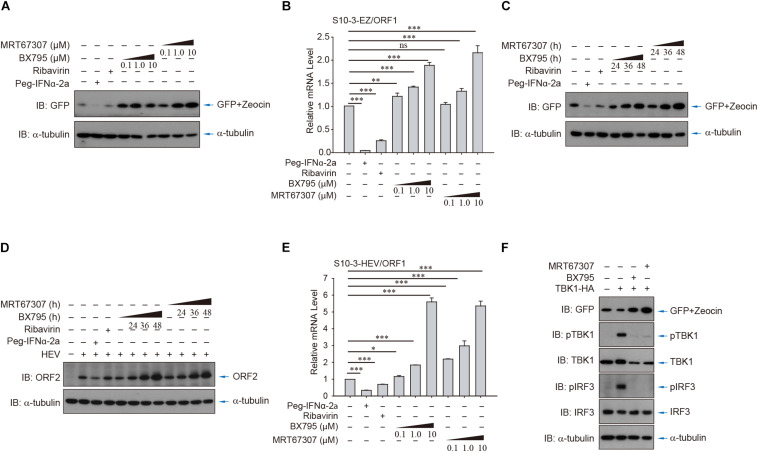
HEV interferes with host antiviral sensing and responses in HEV replicon cells or persistent HEV-infected S10-3 cells. Treatment with TBK1 inhibitors BX795 or MRT67307 in persistent HEV replicon cells S10-3-EZ enhanced GFP expression, in both **(A)** a dose-dependent and **(C)** time-dependent manner. **(B)** In these same cells, BX795 or MRT67307 increased HEV replicon RNA level in a dose-dependent manner. **(D,E)** S10-3 cells stably infected with HEV p6 stock (S10-3-HEV) were established as described (Yin et al., 2017), and expression of **(D)** ORF2 protein or **(E)** ORF1 RNA was determined by western blot or qPCR, respectively. **(F)** Overexpression of TBK1 in persistent HEV replicon S10-3-EZ cells led to the activation of antiviral responses and a decrease in HEV replication. The mean ± SEM is shown, and error bars indicate standard deviation; unless otherwise specified, *n* = 3 independent experiments. Analysis of variance (ANOVA) tests were performed with Bonferroni correction; *P < 0.05; **P < 0.01; and ***P < 0.001.

To further demonstrate the utility of the persistent HEV replicon cell lines, the cells were utilized to evaluate the role of RLR-IRF3 signaling, the innate immune sensing pathway for detection of viral RNA. Two small molecule inhibitors, BX795 and MRT67307, that target the central kinase of RLR-IRF3 signaling, TANK-binding kinase 1 (TBK1), were examined in the HEV replicon S10-3-EZ cells (Figures 6A,B). Intriguingly, inhibition of TBK1 substantially promoted HEV replication in S10-3-EZ cells by WB and qRT-PCR analysis (Figures 6A–C), which were confirmed by quantitative analysis of the enhanced effect of TBK1 inhibitors on HEV replication by FACS in a dose-dependent manner (Supplementary Figures 5A–C) or a time-dependent manner (Supplementary Figures 5D–F). As controls, TBK1 inhibitors did not change the GFP expression level in S10-3-GFP stable cells (Supplementary Figures 5A,D). These results suggest that RLR-IRF3 is important in HEV infection. The S10-3 cells persistently infected with HEV, named S10-3-HEV, were established as described by Yin et al. (2017). The observations in HEV replicon cells above were further confirmed by persistent infection of S10-3 cells with HEV (Figures 6D,E). Furthermore, we demonstrated that activation of RNA innate immune sensing by overexpression of TBK1 in S10-3-EZ cells decreased viral infection (Figure 6F). These data suggest that TBK1 activation is a decisive factor for HEV replication.

**FIGURE 7:**
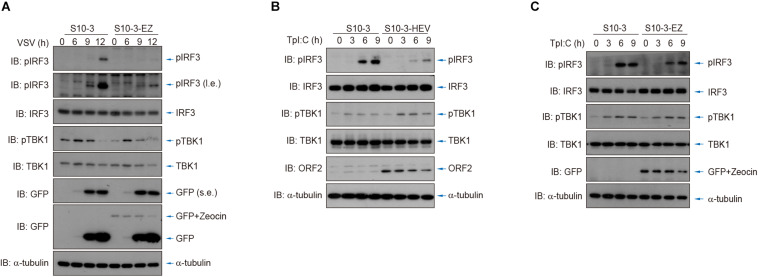
HEV interferes with host antiviral sensing and responses after infection by VSV or transfection of poly(I:C). **(A)** Cytosolic RNA sensing was inhibited in S10-3-EZ compared with the parental S10-3 cells, as determined by IRF3 activation after infection by VSV. **(B,C)** Cytosolic RNA sensing was inhibited in HEV-infected S10-3 cells (S10-3-HEV) compared with mock-infected S10-3 cells **(B)**, or in persistent HEV replicon cell line S10-3-EZ compared with its parental liver cell line S10-3 **(C)**, which was determined by IRF3 activation after transfection of poly(I:C).

Additionally, we used vesicular stomatitis virus (VSV) infection as well as cytosolic exposure to poly(I:C; an RNA analog) to stimulate IRF3 phosphorylation and thereby activate innate RNA sensing. By comparing the persistent HEV replicon cells (S10-3-EZ) or HEV-infected cells (S10-3-HEV) to its parental S10-3 cells, we showed that IRF3 stimulation was severely impeded by HEV replicons or persistent HEV infection (Figure 7). These observations suggest the presence of HEV–host interactions to suppress the host innate immune sensing of viral RNA, an intriguing mechanism that warrants in-depth investigation in the future.

## Discussion

The lack of an efficient cell culture system for HEV has greatly hindered the basic and translational study of HEV. In this study, we successfully developed a persistent HEV cell culture model for genotype 3 human HEV, and subsequently demonstrated the utility of the model in studying antivirals and host innate immune response.

First, we report the establishment of a highly efficient (80% actively HEV-replicating cells), stable and persistent HEV replicon cell culture system, which required only minimal maintenance by zeocin treatment (Figures 1–3). The persistent HEV replicon cell culture avoids the need to constantly transfect cells with viral RNA transcripts and greatly increases the efficiency and reproducibility of HEV replication, which is absolutely critical for HEV antiviral screening. We found that the genotype 3 human HEV replicon replicated efficiently not only in the human hepatoma cell line Huh7-S10-3, but in baby hamster kidney fibroblast cell line BHK-21 as well. This is not particularly surprising, since the genotype 3 human HEV Kernow C-1 strain used in this study is known to replicate in cells from a broad spectrum of species ranging from rodent to primate (Shukla et al., 2011; Shukla et al., 2012), suggesting that the limitation on genotype 3 HEV genome replication in cell culture is not due to a lack of essential translation machinery or cytokines. The p6-EZ persistent HEV replicon in this study lacks both ORF2 and ORF3, which was replaced with an EGFP marker and zeocin resistance gene. Therefore, the life cycle of the persistent HEV replicon p6-EZ is deficient in the steps of uncoating, assembly and extracellular release of progeny virions, but the culture system does release the positive-sense HEV genomic RNA directly into the cytoplasm.

Using a mouse monoclonal antibody against the X domain of HEV ORF1, we detected the endogenous expression of HEV non-structural proteins in persistent HEV replicon cell line BHK-EZ (Figure 2), but not in cells infected with infectious HEV probably due to the limited expression level of ORF1 (data not shown), further illustrating the unique value of these persistent HEV replicon cell cultures for mechanistic study of HEV replication and virus–host interaction in the future.

The values of the replicon cell models in antiviral testing were further assessed. Currently, IFN-α and ribavirin are the only drugs that are available to treat acute and chronic hepatitis E (Todt et al., 2016). We demonstrated that IFN-α was an effective dose-dependent inhibitor of the p6-EZ replicon (Figure 4). Interestingly, the p6-EZ replicon was more sensitive to IFN-α in BHK-21 cells than in Huh-7 cells. Ribavirin is a synthetic guanosine analog that has shown antiviral activity against a range of DNA and RNA viruses (Graci and Cameron, 2006). We also found that ribavirin was also very effective at reducing HEV replication in replicon-bearing cells in a dose-dependent manner in both BHK-21-EZ and S10-3-EZ cells (Supplementary Figure 3). There are two limitations when the replicon model is used to test new antiviral drugs. The efficacy of a potential drug may be underestimated due to the overexpression of ORF1, and only drugs targeting the non-structural proteins can be screened since ORF2 or ORF3 are not expressed. These can be partly overcome by addition of the maximum dosage of the tested drug without obvious cytotoxicity and further verification of the candidate drugs using the animal model.

By using the HEV replicon cell culture system, we demonstrated that the persistent HEV replicon cells suppress the RLR-IRF3 signaling pathway, primarily by abrogation of IRF3 activation (Figures 6,7). It has been reported that HEV infection induced a sustained type III IFN response in infected cells, but the IFN level was insufficient to eliminate the virus (Yin et al., 2017). Since the S10-3-EZ HEV replicon cells possess a complete set of type I IFN signaling, it makes them an ideal cell culture model to study the effect of persistent HEV infection on the RLR-mediated IFN response. A previous study showed that overexpressing of the HEV X domain or the putative cysteine protease domain in HEK293T cells could inhibit IRF3 phosphorylation or have deubiquitinase activity for both RIG-I and TBK-1 (Nan et al., 2014). Consistently, in this study, we found that HEV replication was significantly enhanced when using small molecule inhibitors TBK1 to block the RLR-IRF3 pathway. We also demonstrated, for the first time, that HEV replication is able to inhibit host RLR-IRF3 phosphorylation in the replicon cell line, thus suggesting that HEV has evolved a mechanism to escape the surveillance of innate immunity. Since host innate immune surveillance is closely associated with distinct cell fate and antitumor immunity (Wu et al., 2019) and crosstalk with environmental cues (Zhang et al., 2017), this finding has important implications in understanding HEV pathogenesis. In conclusion, we have generated two cell lines that support persistent replication of HEV replicon p6-EZ genomic and subgenomic RNA. These replicon-bearing cells open the avenues for HEV research and development, particularly in the areas of antiviral screening, persistent HEV infection, and host antiviral sensing and responses.

## Data Availability Statement

The original contributions presented in the study are included in the article/Supplementary Material, further inquiries can be directed to the corresponding authors.

## Author Contributions

L-DX, FZ, PX, and Y-WH designed the research, analyzed the data, and wrote the manuscript. L-DX, FZ, LP, W-TL, CC, and Y-WH performed the research. All authors contributed to the article and approved the submitted version.

## Conflict of Interest

The authors declare that the research was conducted in the absence of any commercial or financial relationships that could be construed as a potential conflict of interest.
