# Supplementary material for: Stable Expression of a Hepatitis E Virus (HEV) RNA Replicon in Two Mammalian Cell Lines to Assess Mechanism of Innate Immunity and Antiviral Response
Source: Front Microbiol. 2020 Dec 3;11:603699. doi: 10.3389/fmicb.2020.603699 (PMC7793998; doi:10.3389/fmicb.2020.603699)
Supplement: Supplementary file 1 [file Data_Sheet_1.docx]

**Supplementary Information for**

**Title： Stable expression of a hepatitis E virus (HEV) RNA replicon in two mammalian cell lines as a model system to assess mechanism of innate immunity and antiviral response**

**Authors：**Ling-Dong Xu, Fei Zhang, Lei Peng, Wen-Ting Luo, Chu Chen, Pinglong Xu, Yao-Wei Huang

The corresponding authors:

Pinglong Xu, Email: [xupl@zju.edu.cn](mailto:xupl@zju.edu.cn)

Yao-Wei Huang, Email: [yhuang@zju.edu.cn](mailto:yhuang@zju.edu.cn)

**This file includes:**

Supplemental Methods

Figures S1 to S5

***SI Appendix***

**Supplementary Methods**

**Real-time RT-PCR assays.**

The cells were counted to be 100,000 cells in each sample before collection. The total RNA was extracted from the HEV replicon cells, using Trizol reagent (Thermo), according to the manufacturer's instructions. HEV replicon RNA titers were determined by one-step qRT-PCR targeting the ORF1 of HEV with the primers 5ʹ-GCCTGGAGCAAGACATTCTG-3′ and 5ʹ-AGCAAGCACTGACTCCTCAT-3′ and the probe 6-carboxyfluorescein (FAM)-TGGCCCGTGGTTCCGTGCCA (TAMRA). The standard curve was generated by diluting a known number of full-length RNA of HEV replicon to allow absolute quantitation of HEV replicon RNA copy numbers.

The other descriptions of the materials and methods used for the Supplementary Figures are provided in the main text.

**
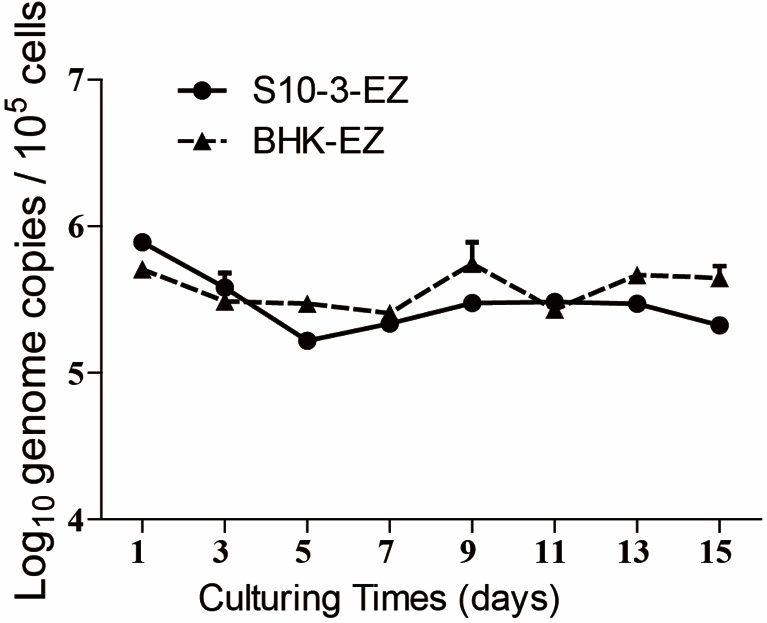
Fig. S1. Detection of HEV replicon stability in persistent HEV replicon cell lines BHK-EZ and S10-3-EZ by qRT-PCR.**

**
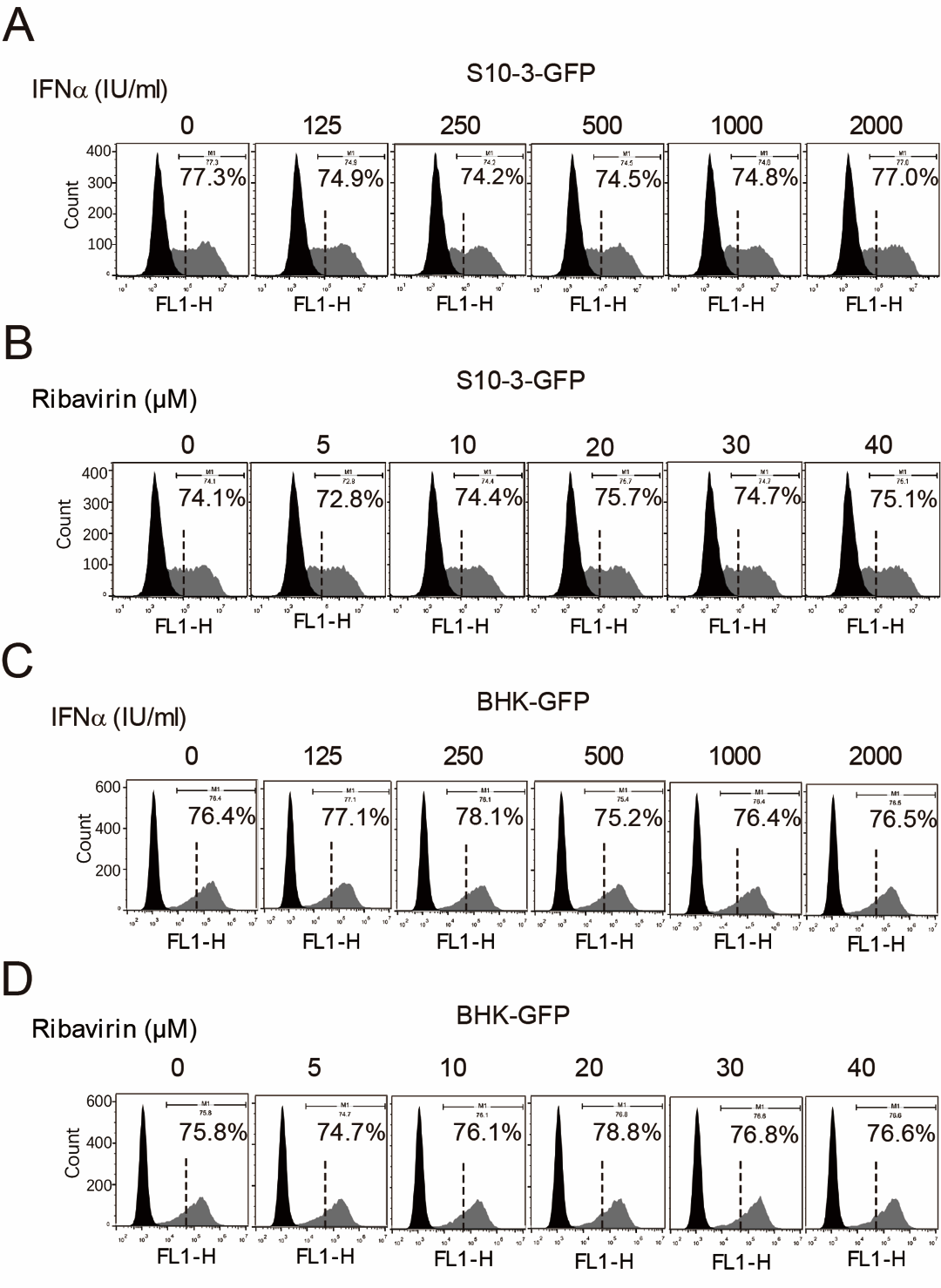
Fig. S2. The effects of IFNα and ribavirin on stable cell lines S10-3-GFP and BHK-GFP.**

(A, B) S10-3-GFP were treated with IFNα and ribavirin, GFP-positive cells were counted by FACS. (C, D) BHK-GFP were treated with IFNα and ribavirin, GFP-positive cells were counted by FACS.

**
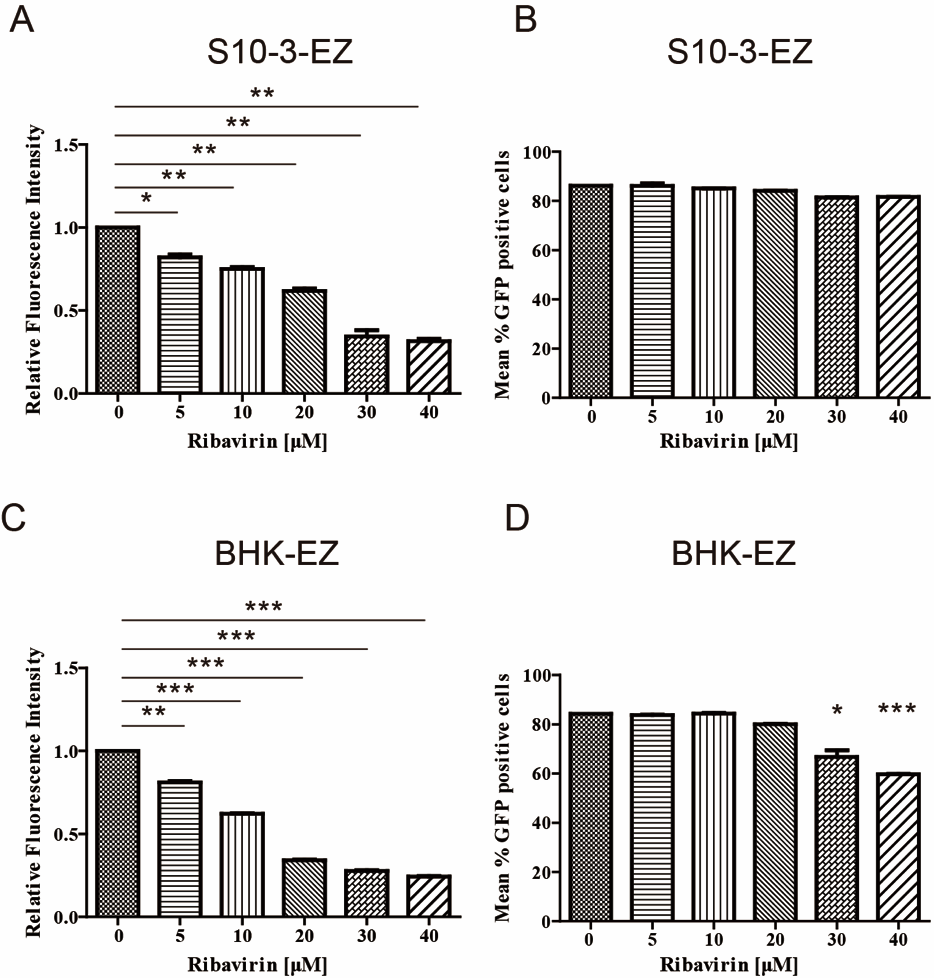
Fig. S3. The effects of ribavirin on persistent HEV replicon cell lines.**

The effect of ribavirin treatment on **(A, B)** S10-3-EZ and **(C, D)** BHK-EZ replicon cell lines was determined. (**A, C**) Quantitative FACS analysis confirming ribavirin inhibition of GFP expression by relative fluorescence intensity (RFI). (**B, D**) The number of GFP-positive cells as determined by FACS after ribavirin treatment is shown as a percentage of total counted cells.

**
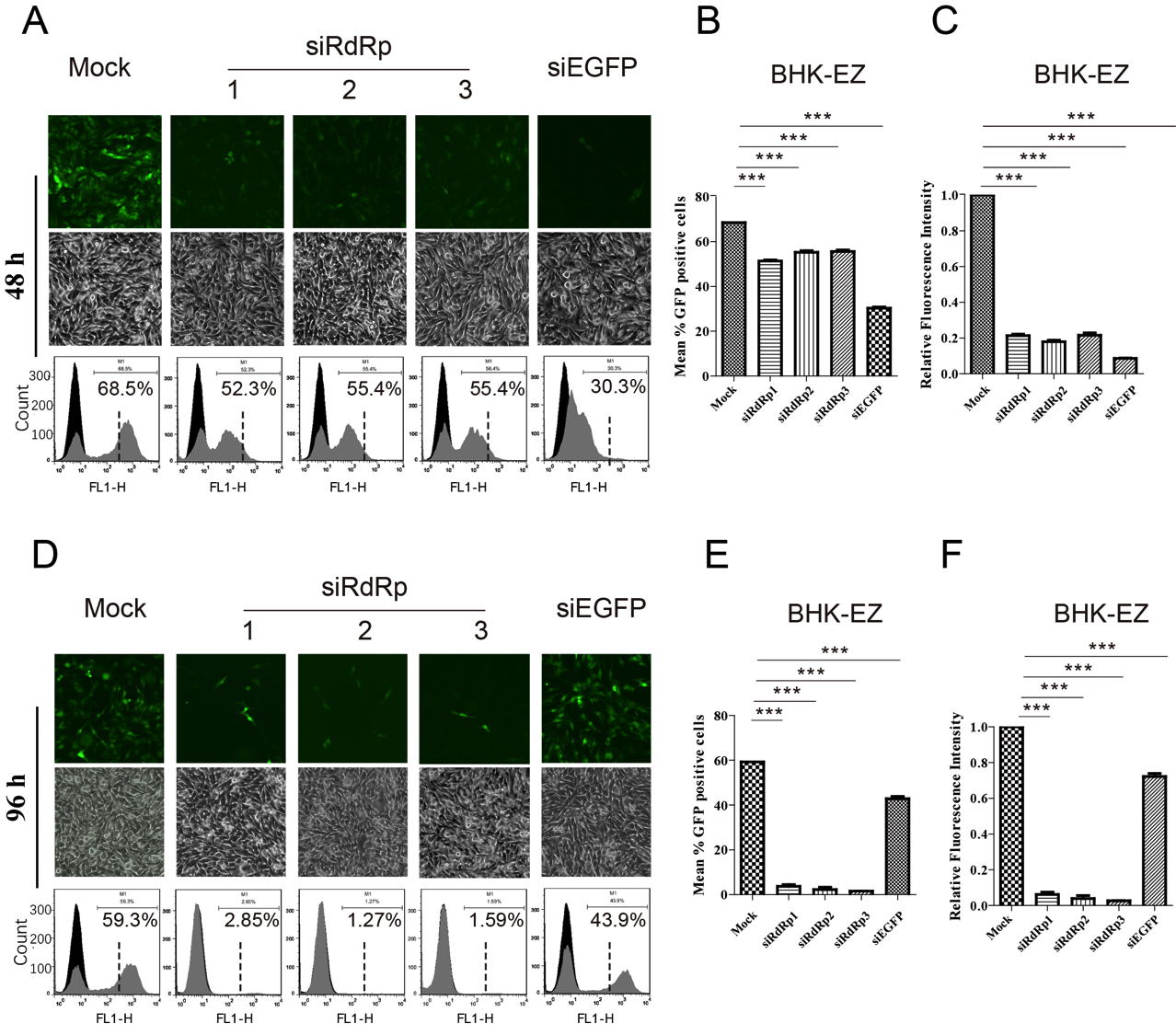
Fig. S4. The effects of siRNA interference on persistent HEV replicon cell line BHK-EZ.**

**(A, D)** BHK-EZ cells were observed by fluorescence microscopy, and GFP-positive cells were counted by FACS 48 or 96 h after siRNA treatment. **(B, E)** The number of GFP-positive cells as determined by FACS is shown as a percentage of total counted cells (hpt: hours post-transfection). **(C, F)** Quantitative FACS analysis confirming siRNA inhibition of GFP expression by RFI. GFP expression in cells treated with siRNA is shown as a relative percentage of the mock-treated control. Error bars indicate standard deviation; **P*<0.05; ***P*<0.01; and ****P*<0.001.

**
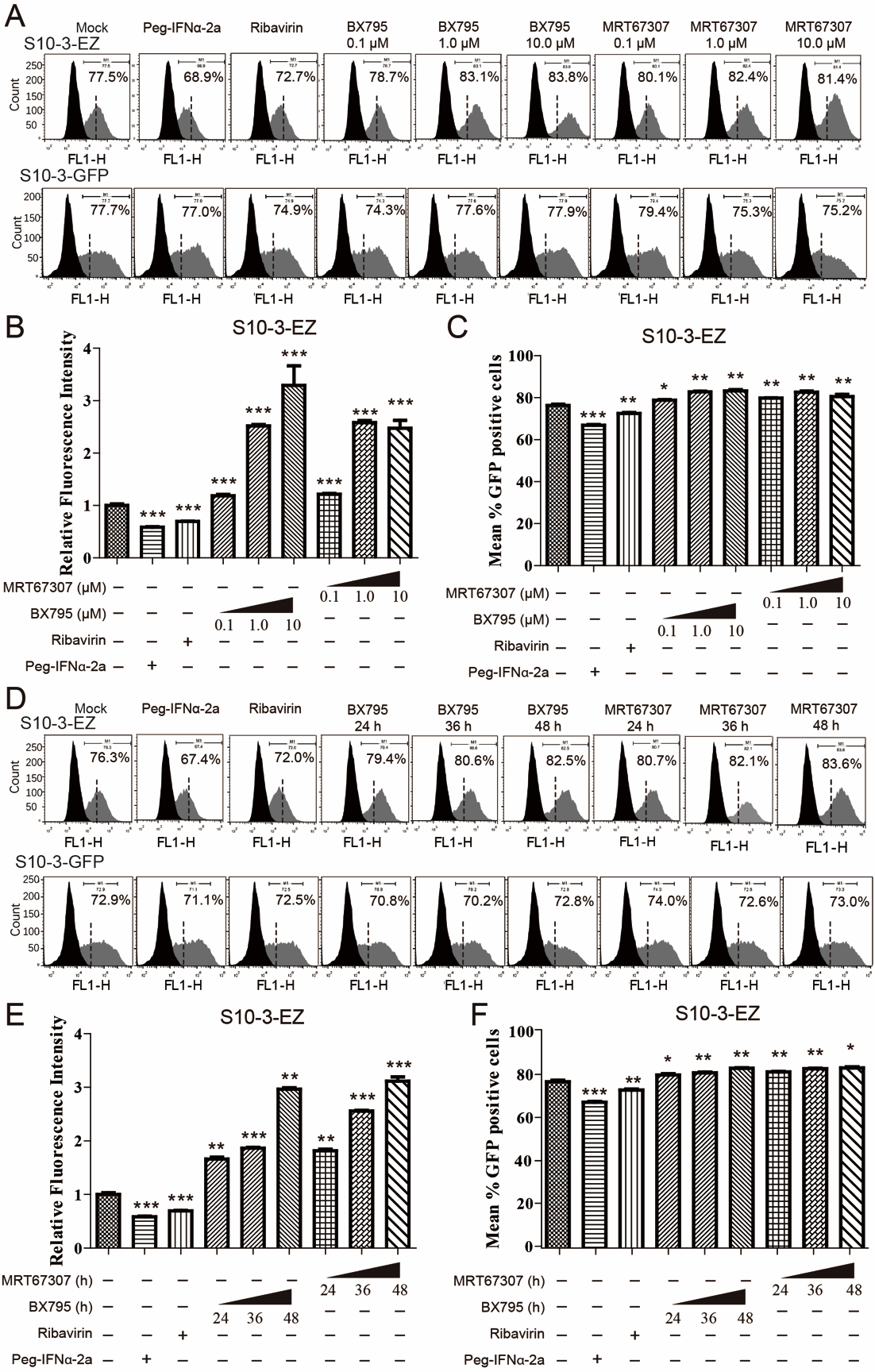
Fig. S5. Treatment with TBK1 inhibitors BX795 or MRT67307 in persistent HEV replicon cells S10-3-EZ enhanced GFP expression.**

Treatment with TBK1 inhibitors BX795 or MRT67307 in persistent HEV replicon cells S10-3-EZ enhanced GFP expression, in both **(A)** a dose-dependent and **(D)** time-dependent manner. **(B, E)** Quantitative FACS analysis confirming treatment with TBK1 inhibitors of GFP expression by RFI. **(C, F)** The number of GFP-positive cells as determined by FACS is shown as a percentage of total counted cells. GFP expression in cells treated with TBK1 inhibitors is shown as a relative percentage of the mock-treated control. Error bars indicate standard deviation; **P*<0.05; ***P*<0.01; and ****P*<0.001.
